# Macro‐ and microcirculatory factors affecting foot temperature change during walking

**DOI:** 10.1113/EP093712

**Published:** 2026-07-29

**Authors:** Jose G. Anguiano‐Hernandez, Jenna K. Burnett, Michael F. Allen, Cody P. Anderson, Kota Z. Takahashi, Song‐Young Park

**Affiliations:** ^1^ Department of Health and Kinesiology University of Utah Salt Lake City Utah USA; ^2^ School of Health and Kinesiology University of Nebraska at Omaha Omaha Nebraska USA; ^3^ Department of Biomedical Engineering University of Utah Salt Lake City Utah USA; ^4^ Department of Physical Medicine & Rehabilitation University of Utah Salt Lake City Utah USA; ^5^ Department of Cellular and Integrative Physiology University of Nebraska Medical Center Omaha Nebraska USA

**Keywords:** foot temperature change, gradient walking, leg blood flow, leg muscle oxygen consumption

## Abstract

Change in skin temperature is a complex process affected by several factors. During walking, increased microcirculatory activity, such as muscle metabolism, could increase foot temperature. The ensuing macrocirculatory responses, such as increased leg blood flow (LBF), might also increase foot temperature. However, it is unclear whether there is a relationship between these physiological measures and foot temperature during walking. Here, we examined the relationships among macrocicrulation (e.g., LBF), microcirculation [e.g., tissue saturation index (TSI)] and the change in foot temperature during walking on different slopes. Healthy young adults (*n *= 21) walked on a 0°, +5° and −5° slope in random order for 10 min at 1.2 m/s. Before and after each walking bout, LBF, TSI and foot temperature were measured on the dominant leg of the participant. TSI was measured at the vastus lateralis (VL), medial gastrocnemius (MG) and dorsal foot. Uphill walking caused a greater increase in LBF (*P *< 0.001) and greater reductions in TSI for VL (*P *< 0.001) and MG (*P *< 0.027) in comparison to level or downhill walking, probably reflecting the increased muscle oxygen demand. Only changes in dorsal foot TSI were positively associated with foot temperature change (β = 0.064, *P *= 0.026), whereas LBF and TSI for VL and MG showed no significant associations (*P *> 0.192). These results suggest that the macrocirculation increases to accommodate the metabolic demands of leg muscles, without affecting foot skin temperature change. We speculate that foot temperature change might be affected largely by mechanisms not tested in our study, such as cutaneous blood flow, sweating and convective heat loss, which should be investigated in the future.

## Abstract (highlights)

- **What is the central question of the study?**
  How do the macrocirculation and microcirculation affect foot skin temperature change?
- **What is the main finding and its importance?**
  High plantar temperature increases the risk of foot ulceration in cardiovascular disease patients, although the mechanisms behind increased foot temperature are unclear. Using Doppler ultrasound and near‐infrared spectroscopy, we showed that changes in macrocirculation do not influence foot temperature change, but changes in microcirculation at the foot do. These findings elucidate potential mechanisms driving an increase in foot temperature, which can later establish therapeutic targets for the prevention of foot ulceration in those with cardiovascular diseases.

## 1 INTRODUCTION

Skin temperature is an important biomarker for assessing the risk of tissue damage (van Netten et al., 2014; Yavuz & Davis, 2010; Yavuz et al., 2015). Activities of daily living, such as walking, can increase the plantar skin temperature via friction between the bottom of the foot and the ground (i.e., shear forces) and metabolic processes. The resulting increase in temperature during walking (Burnett et al., 2025; Reddy et al., 2017) or running (Shimazaki & Murata, 2015) can predispose tissue on the plantar surface of the foot to several forms of breakdown, such as blisters (Yavuz & Davis, 2010), calluses (Yavuz et al., 2015) and foot ulcers in those with diabetes mellitus (D. G. Armstrong et al., 2017; van Netten et al., 2013; Waaijman et al., 2014; Yavuz et al., 2015, 2019) and peripheral artery disease (Hinchliffe et al., 2020). However, little is known about the interplay between biomechanical factors and the local vascular circulation, and their effects on foot plantar skin temperature change.

Shear forces on the plantar surface of the foot could be one factor influencing plantar temperature increase. Previous research found a moderate positive correlation between shear forces and foot temperature increase in healthy young adults (Yavuz et al., 2014). Gonzalez et al. (2021) solidified this relationship across walking tasks with varying shear force demands. They reported a positive correlation between shear forces and temperature change in the forefoot during toe walking in circles of different radii (Gonzalez et al., 2021). Other research has shown that shod walking can elicit a temperature increase of ∼4.6°C in healthy adults between 30 and 40 years old and an increase of ∼5.5°C in adults >40 years of age (Reddy et al., 2017). Running amplifies the temperature responses, because an average increase of ∼6°C has been reported underneath the hallux during running (Shimazaki & Murata, 2015). These studies highlight the influence of biomechanical factors in foot temperature responses, whereas studies on clinical populations highlight the potential role of other factors, such as vascular physiology, on foot temperature change. For example, patients with diabetes have altered blood flow to the lower limbs (Adam et al., 2017) and have higher resting (Renero‐C, 2017) and post‐walking (Najafi et al., 2012) plantar temperature profiles compared with healthy counterparts. Therefore, vascular physiology could influence changes in plantar temperature concurrently with biomechanical factors.

Macrocirculatory function, such as blood flow, could play a role in plantar skin temperature change, as large conduit arteries deliver oxygenated blood throughout the body to maintain tissue health. Changes in leg blood flow (LBF) can be influenced by lower limb movement (Ratchford et al., 2019; Rossman et al., 2015). Previous research has used Doppler ultrasound to demonstrate this process using in vivo analyses during knee‐extension tasks (Ratchford et al., 2019) and cycling (Rossman et al., 2015). Results suggest that submaximal knee‐extension exercise of 40% maximum workload can significantly increase LBF compared with resting blood flow rates, with significantly greater amounts of LBF occurring at 60% and 80% maximum workload (Ratchford et al., 2019). A similar approach has been used to investigate LBF during cycling, showing that increasing cycling intensity can increase the blood flow rate in the femoral artery (Rossman et al., 2015). These results suggest that increased physical activity influences the amount of blood flow to the lower limbs. However, it is unclear how LBF changes during walking at different intensities and how these changes would affect foot temperature change.

Changes in leg macrocirculatory function will probably be accompanied by changes in the microcirculation, such as the leg muscle tissue saturation index (TSI) (Bopp et al., 2014), which is the proportion of the haemoglobin/myoglobin within a tissue that is bound to oxygen. Larger conduit arteries bifurcate into feed arteries, smaller arterioles and capillaries in human tissue to deliver oxygenated blood to the muscles and skin (Klabunde, 2022). Previous research has reported that higher walking speeds decreased muscle oxygenation levels in the vastus lateralis (VL) and lateral gastrocnemius muscles relative to pre‐walking measurements in humans (Hiroyuki et al., 2002), suggesting that oxygen consumption in muscle tissue increases with the intensity of locomotion. In guinea fowl, different muscles of the legs have different metabolic cost profiles during locomotion, as shown by differences in skeletal muscle blood flow during running, using a biofluorescent microsphere technique (Rubenson et al., 2006). However, skeletal muscle tissue oxygen transport and utilization have yet to be studied in conjunction with LBF and plantar temperature increase during walking in humans, creating a gap in knowledge regarding the mechanisms of foot temperature change during walking.

The purpose of this study was to examine the changes in LBF, leg muscle TSI and plantar surface temperature during walking on different slopes (level, incline and decline). We chose to compare walking on different slopes because it influences the metabolic demand of walking (Abe et al., 2015; Minetti et al., 2002). We hypothesise that incline walking will elicit the greatest increase in LBF and plantar surface temperature and the greatest reduction in leg muscle TSI relative to pre‐walking, owing to the greatest oxygen demands in the lower limbs at increased walking intensities (Hiroyuki et al., 2002). We also hypothesise that changes in LBF will be positively correlated with plantar temperature change, and changes in leg muscle TSI will be negatively correlated with plantar temperature change during walking.

## 2 MATERIALS AND METHODS

### 2.1 Ethical approval

The study followed the ethical standards of the *Declaration of Helsinki*, except for registration in a database. Informed consent from all participants was obtained in writing, and all procedures were approved (155345) by the Institutional Review Board of the University of Utah.

### 2.2 Participants

Twenty‐one healthy young adults (11 females, 26.6 ± 3.9 years old, 69.3 ± 9.3 kg, 1.7 ± 0.1 m) were recruited for this study. Exclusion criteria included having a leg injury or fracture within the last 6 months, a neurological disorder of the legs, taking medication that causes dizziness, needing an assistive device to walk, or cardiovascular disease (e.g. peripheral artery disease or diabetes). Participants were instructed to avoid alcoholic beverages, smoking, caffeine, large meals, ointments, cosmetics, showering and sunbathing for ≥4 h before they visited the laboratory (Moreira et al., 2017). We did not explicitly screen participants undergoing hormonal therapy.

### 2.3 Experimental protocol

Data collection took place during a single visit to the laboratory. Participants donned standardized footwear and custom‐made compression shorts in appropriate sizes for each participant, supplied by the laboratory prior to data collection. The shorts had small openings over the inguinal area on both legs that allowed investigators to place an ultrasound probe over the dominant‐side common femoral artery. Participants were also given standardized open‐toe shoes (Genesis Sandal, Xero Shoes, Broomfield, CO, USA) to wear throughout the data collection. Thermistors (Type MA—Biomedical Chip Thermistors, Amphenol Sensors, St Marys, PA, USA) were taped over eight sites on each foot: plantar hallux, dorsal hallux, first metatarsophalangeal joint (MTP), second MTP, fifth MTP, medial arch, lateral arch and heel. Near‐infrared spectroscopy (NIRS) probes were placed on the VL, medial gastrocnemius (MG) and dorsal aspect of the foot (DF) on the dominant leg of the participant. Neoprene was wrapped over each NIRS probe to prevent ambient light from affecting measurements. An ultrasound probe (uSmart 3300 NexGen and 15L4A Linear Transducer, Terason, Burlington, MA, USA) was placed over the common femoral artery of the dominant leg of the participant to measure femoral artery diameter and blood flow velocity at 30 Hz. The probe was fixed to the participant using a custom‐made three‐dimensional (3D) printed bracket and athletic tape. The interior aspect of the bracket was shaped to fit the ultrasound probe, ensuring a snug fit and minimal movement inside the bracket.

Prior to each walking condition, participants rested seated for ≥15 min, with their legs extended and supported, in order that plantar skin could reach a stable temperature. Skin temperature was monitored in real time during this period to ensure that a stable temperature was reached. After resting, a 1 min baseline measurement of femoral artery diameter, blood flow velocity, NIRS data and foot temperature were recorded while participants were standing quietly. This baseline measurement was done within 5 min of the participant standing from their resting position. Participants then walked on an instrumented treadmill (FIT5, Bertec, Columbus, OH, USA) at 1.2 m/s on a 0° slope for 10 min, followed by a −5° or +5° slope for the same speed and duration in random order. We chose a walking time of 10 min to determine the acute effects of local circulatory changes on foot skin temperature without meaningfully increasing core temperature, which would contribute to the increase in foot skin temperature. A 5 min post‐walking measurement of femoral artery diameter, blood flow velocity, NIRS signals and foot temperature was recorded after each walking condition while participants were standing quietly.

### 2.4 Ultrasound

A Doppler ultrasound‐based method previously developed in our laboratory (Anguiano‐Hernandez et al., 2025) was used to measure femoral artery diameter and blood flow velocity in the dominant leg at 30 Hz. The ultrasound transducer was fastened over the common femoral artery using a 3D‐printed bracket shaped explicitly for the probe. Athletic tape was wrapped around the waist to hold the probe in place. The depth of the B‐mode image was adjusted such that the upper and lower artery walls were visible in the frame, typically either 3 or 4 cm. The blood flow velocity graph was set to its greatest range after the depth was set, with the lower and upper bounds of the velocity graph ranging from −60 to 240 cm/s, respectively. The insonation angle of the ultrasound beam was kept at 60° (Logason et al., 2001). Settings were adjusted as needed for each participant but kept consistent across all walking conditions.

#### 2.4.1 TSI

TSI was measured at the dominant‐leg VL, MG and DF using NIRS probes and Oxysoft (Artinis, The Netherlands) at 100 Hz. The NIRS sensors use spatially resolved spectroscopy (SRS) to determine the TSI of the tissue using Equation 1, which has previously been used to quantify the TSI of skeletal muscle tissue during walking (Hiroyuki et al., 2002; Miura et al., 2000; Pekas et al., 2021).

(1) $$\mathrm{TSI}=\frac{\left[O_{2}\mathrm{Hb}\right]}{\left[\mathrm{HHb}\right]+\left[O_{2}\mathrm{Hb}\right]}\times 100$$

where [O_2_Hb] is the oxyhaemoglobin concentration and [HHb] the deoxyhaemoglobin concentration.

We chose to measure tissue oxygenation at the VL because it is the largest muscle of the quadriceps (Bordoni & Varacallo, 2023b; Khan & Arain, 2023). Likewise, we chose the MG because it is the largest of the superficial muscles of the triceps surae (Bordoni & Varacallo, 2023a). We chose to measure tissue oxygenation at the DF because that site allows for total contact between the skin and the NIRS probe.

### 2.5 Plantar temperature

Eight thermistors (Type MA—Biomedical Chip Thermistors, Amphenol Sensors, St Marys, PA, USA) were used to measure skin temperature at eight sites of each foot at 1 Hz, as described in section 2.3. The thermistors measure the change in the resistance inside the probe and calculate a temperature based on the change in resistance.

### 2.6 Data analysis

Ultrasound videos were trimmed into 1 min baseline and 5 min post‐walking videos. Trimmed ultrasound videos were analysed using FloWaveUS, a previously validated open‐source software package (Coolbaugh et al., 2016). FloWaveUS works by digitizing the B‐mode image and blood flow velocity graph from a screen recording of the duplex mode ultrasound. Instantaneous arterial diameter was extracted from videos by calculating pixel distances from the centre of the artery lumen and an artery wall. Those pixel distances were then converted to centimetres. A similar method was used to extract instantaneous blood flow velocity. Instantaneous arterial diameter and blood flow velocity were exported from FloWaveUS at 100 Hz. Raw arterial diameter data were filtered using a second‐order recursive Butterworth filter with a 10 Hz cut‐off frequency. We chose a Butterworth filter because the noise in the arterial diameter data presented more cyclically. A 10 Hz cut‐off frequency was chosen because a residual analysis was done using Winter's method (Winter, 2009). Briefly, a range of cut‐off frequencies were tested, and the lowest cut‐off frequency that did not cause large residuals between the raw and filtered data was chosen for our analysis. Raw blood flow velocity data were filtered using a fifth‐order one‐dimensional median filter. We chose to use a median filter because the noise in the blood flow velocity data presented more randomly throughout the recording. Instantaneous LBF was calculated (in millilitres per minute) using Equation 2:

(2) $$\mathrm{LBF}=V_{\mathrm{BF}}\times \pi \times {\left(\frac{D_{\mathrm{FA}}}{2}\right)}^{2}\times 60$$

where *V*_BF_ is the blood flow velocity (in centimetres per second) and *D*_FA_ is femoral artery diameter (in centimetres). LBF was then converted to litres per minute.

Foot temperature was calculated as the mean temperature of each thermistor placed on the foot. Temperature data from the participant's dominant foot were used for analysis.

Pre‐walking baselines (30 s) of arterial diameter, blood flow velocity, LBF, TSI (of VL, MG and DF) and foot temperature were calculated as the means of all data points in the last 30 s of the baseline time series data. Post‐walking means of all variables were calculated as the means of all data points in the first 30 s of post‐walking time series data. Changes in each variable were calculated as the difference between the post‐walking mean and the baseline mean. All data analysis was done using MATLAB (R2023a, MathWorks, Natick, MA, USA).

### 2.7 Statistical analysis

Statistical analysis was done in SPSS v.30 (IBM, Armonk, NY, USA). Separate one‐way repeated‐measures ANOVAs were used to test the main effect of slope on each outcome variable. The assumption of sphericity was checked using Mauchly's test for sphericity to compare the variance of our outcome variables in each condition. Data that did not pass the assumption of sphericity were corrected using Greenhouse–Geisser corrections. Significant main effects of slope were investigated further using Student's paired *t*‐tests with Bonferroni corrections.

Linear mixed‐effects models (LMMs) were used to investigate the relationship between fixed effects (walking conditions, changes in LBF, changes in leg muscle TSI, baseline foot temperature, ambient temperature and ambient humidity) and foot temperature change (outcome variable). The first LMM with fixed effects of walking condition (Level condition as the referent category), ambient temperature and ambient humidity was done to determine their relationship to foot temperature change. Fixed effects that were not significantly associated with foot temperature change were not included in subsequent models to avoid overfitting the model. Given that changes in LBF and changes in leg muscle TSI were expected to be correlated, they were divided into separate models to account for collinearity. The second model included baseline foot temperature and changes in LBF as fixed effects, and the third model included baseline foot temperature and changes in VL, MG and DF TSI as fixed effects. All statistical analyses were done at α = 0.05.

## 3 RESULTS

### 3.1 Foot temperature

**FIGURE 1:**
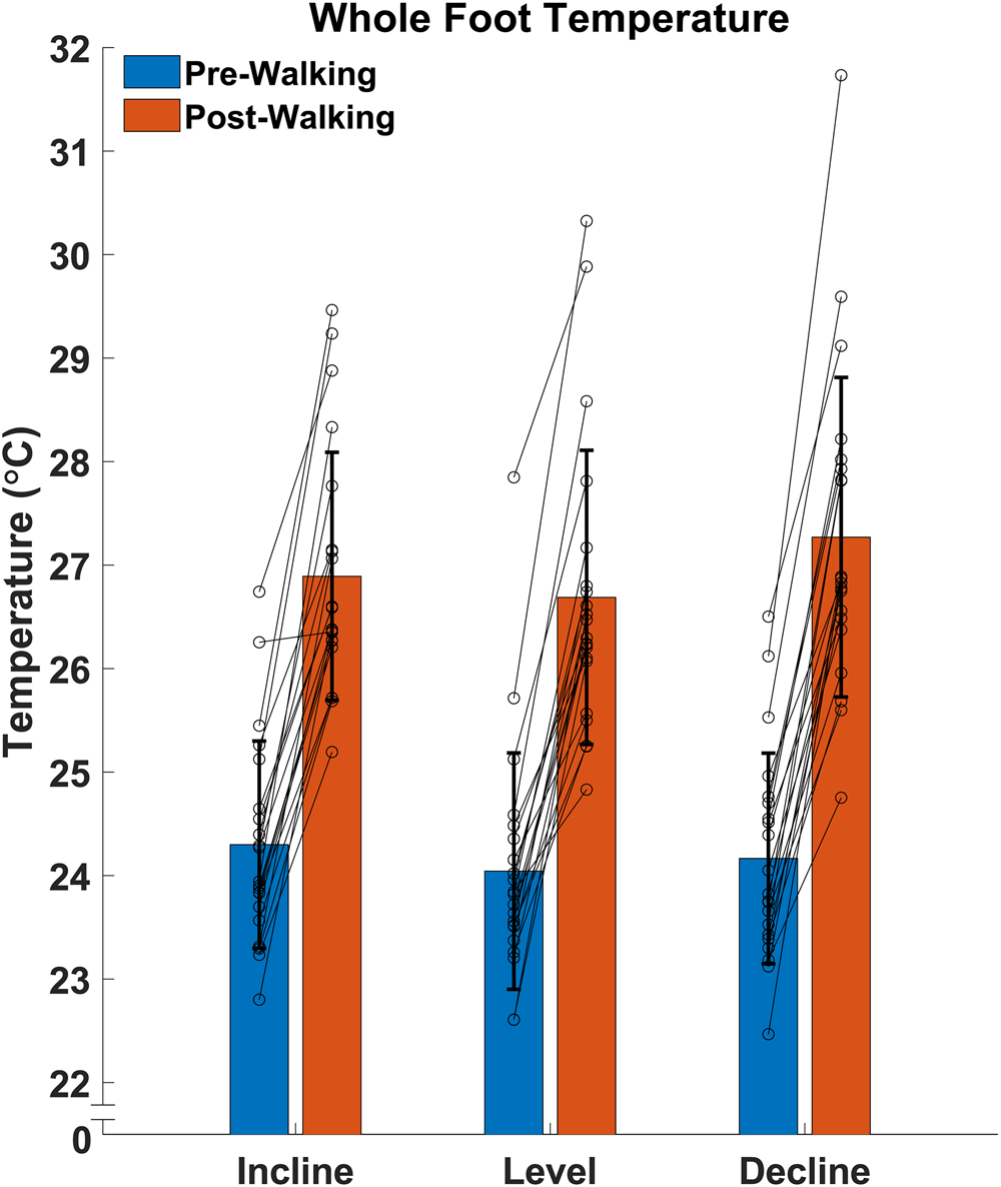
Whole foot temperature before (blue) and after (orange) walking. There was no significant main effect of slope on the change in foot temperature from baseline (*P* = 0.296), suggesting that the increase in foot temperature was similar in all conditions. Connected dots are individual participant data.

One‐way repeated‐measures ANOVA did not show a significant main effect of slope on foot temperature change across conditions (*P* = 0.281, η^2^_partial_ = 0.105), suggesting that walking on different slopes did not affect changes in foot temperature (Figure 1).

### 3.2 LBF

**FIGURE 2:**
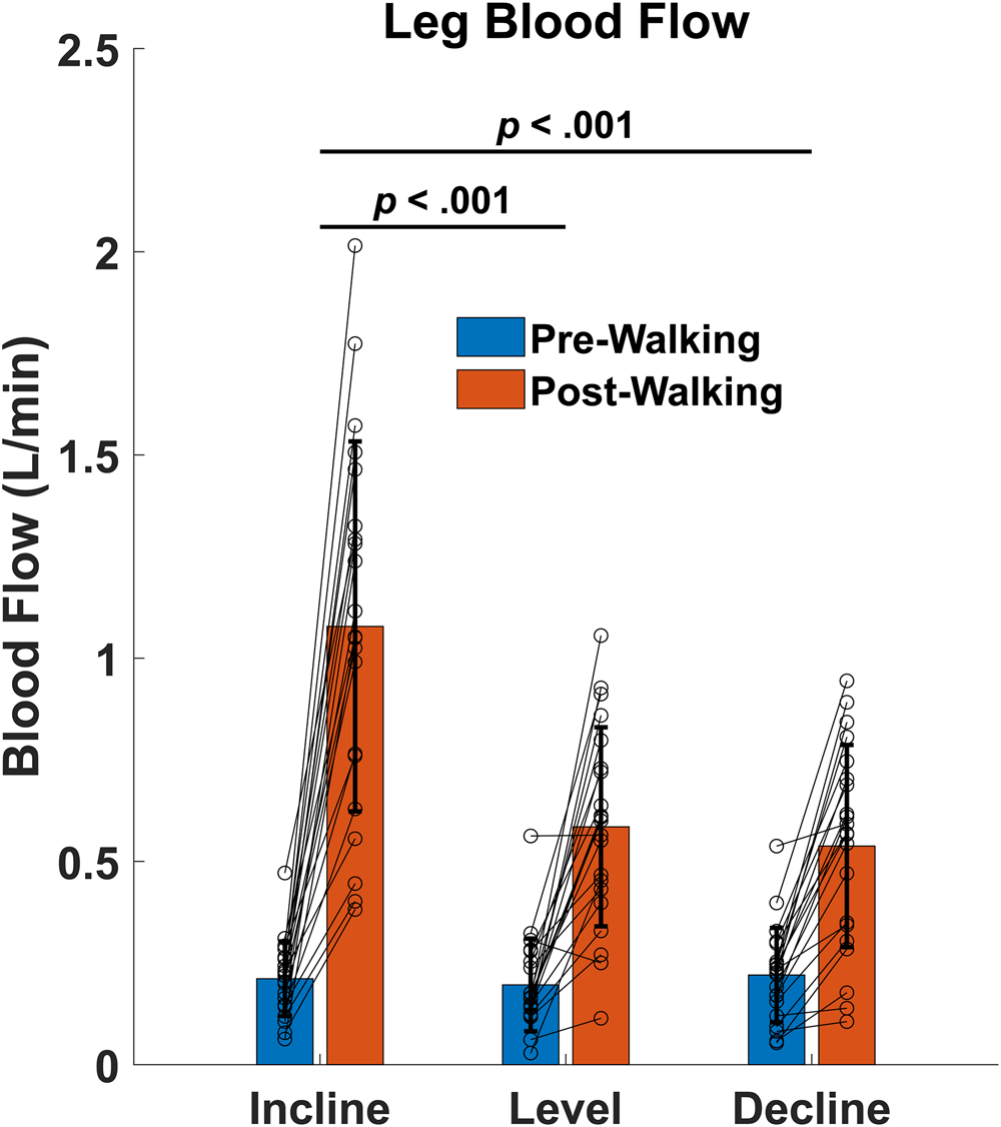
Leg blood flow (LBF) before (blue) and after (orange) walking. There was a significant main effect of walking slope on LBF change from baseline (*P* < 0.001), with uphill walking causing the greatest increase in LBF in comparison to level walking (*P* < 0.001) and decline walking (*P* < 0.001). These results suggest that uphill walking causes a greater increase in LBF in comparison to walking on a level or downhill slope.

There was a significant main effect of slope on the change in LBF (*P* < 0.001, η^2^_partial_ = 0.625). Bonferroni‐corrected pairwise comparisons showed that the increase in LBF was greater in the Incline condition compared with the Level condition (mean difference: 0.48 L/min, *P* < 0.001) and the Decline condition (mean difference:.55 L/min, *P* < 0.001), suggesting that walking uphill caused the greatest increase in LBF compared with level and downhill walking (Figure 2).

### 3.3 TSI

**FIGURE 3:**
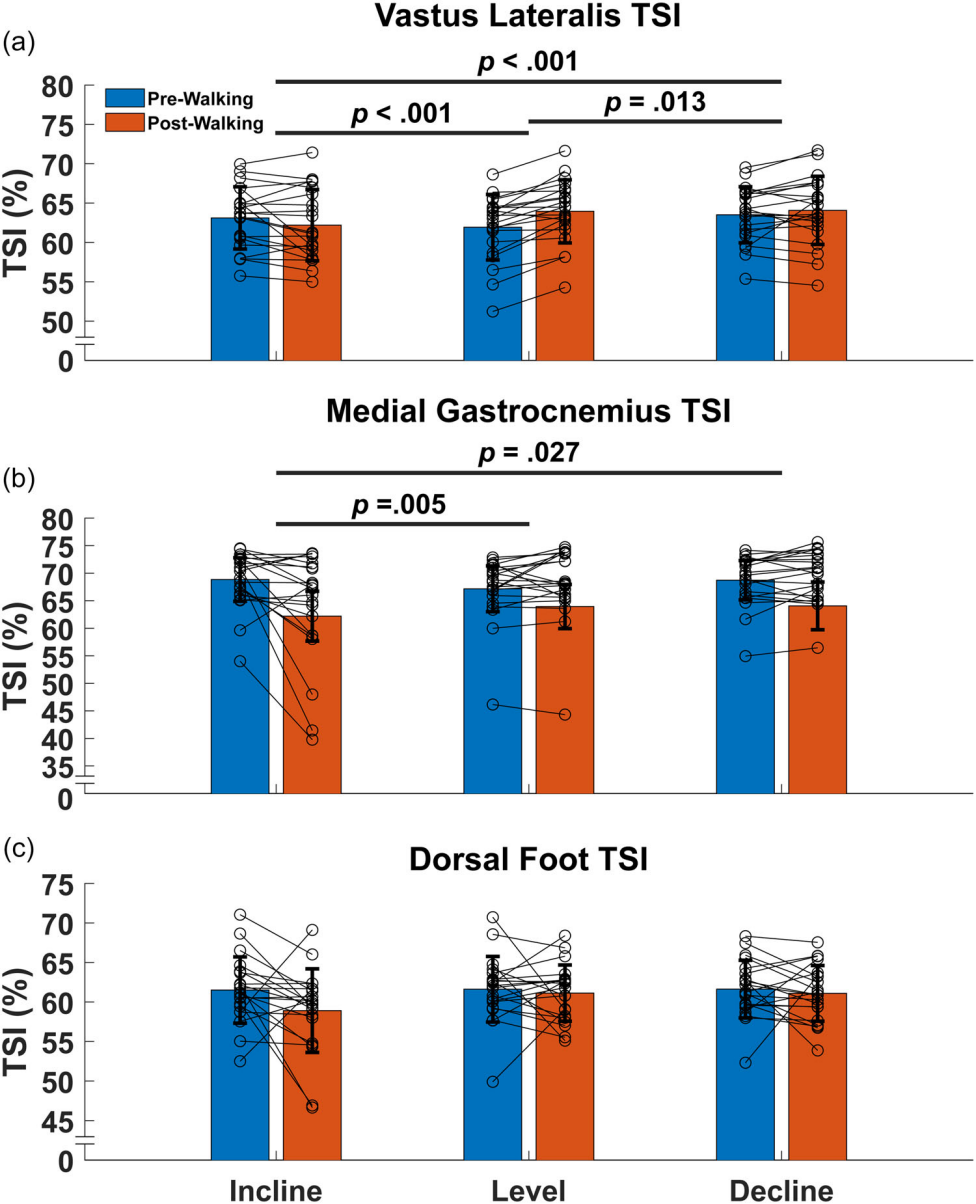
Tissue saturation index (TSI) before (blue) and after (orange) walking in the vastus lateralis (a), medial gastrocnemius (b) and dorsal foot (c). There was a significant main effect of walking slope on the change in vastus lateralis TSI from baseline (*P* < 0.001), with uphill walking causing the greatest decrease in vastus lateralis TSI compared with the Level (*P* < 0.001) and Decline conditions (*P* = 0.013). The Decline condition also caused a greater decrease in vastus lateralis TSI compared with the Level condition (*P* = 0.006). Walking slope also significantly affected the change in medial gastrocnemius TSI from baseline (*P* = 0.004), with Incline uphill walking causing a greater decrease in medial gastrocnemius TSI compared with Level (*P* = 0.005) and Decline walking (*P* = 0.027).

There was a significant main effect of slope on the change in VL TSI across walking conditions (*P* < 0.001, η^2^_partial_ = 0.514). Bonferroni‐corrected pairwise comparisons showed that the Incline condition caused a greater decrease in VL TSI compared with the Level (mean difference: −2.94%, *P* < 0.001) and Decline conditions (mean difference: −1.5%, *P* = 0.013). Additionally, the Decline condition also caused a greater decrease in VL TSI compared with the Level condition (mean difference: −1.47%, *P* = 0.006). These results suggest that oxygen consumption in the VL muscle is greatest when walking uphill and lowest when walking on a level surface (Figure 3).

There was a significant main effect of slope on the change in MG TSI (*P* = 0.004, η^2^_partial_ = 0.322). Bonferroni‐corrected pairwise comparisons showed the decrease in MG TSI was greater in the Incline condition compared with the Level (mean difference: −5.79%, *P *= 0.005) and Decline conditions (mean difference: −5.68%, *P *= 0.027). This result suggests that walking uphill caused greater oxygen consumption in the MG muscle compared with walking on a level or downhill surface (Figure 3).

There was no significant main effect of slope on the change in DF TSI (*P* = 0.085, η^2^_partial_ = 0.127), suggesting that walking slope did not affect the change in DF TSI (Figure 3).

### 3.4 Association between physiological measures and plantar temperature

The first LMM showed that walking condition, ambient temperature and ambient humidity did not contribute significantly to changes in foot temperature (Table 1). Thus, these fixed effects were removed to simplify further models.

**TABLE 1:** Estimates of the influence of walking slope, ambient temperature and ambient humidity on changes in foot temperature.

Predictor	Regression coefficient	*P*‐value	95% Confidence interval
Intercept	2.934	0.405	−4.134 to 10.003
Incline slope	−0.085	0.752	−0.624 to 0.454
Decline slope	0.418	0.126	−0.122 to 0.958
Level slope	–	–	–
Ambient temperature	−0.040	0.778	−0.323 to 0.244
Ambient humidity	0.027	0.330	−0.029 to 0.083

Dependent variable: Δfoot temperature.

**FIGURE 4:**
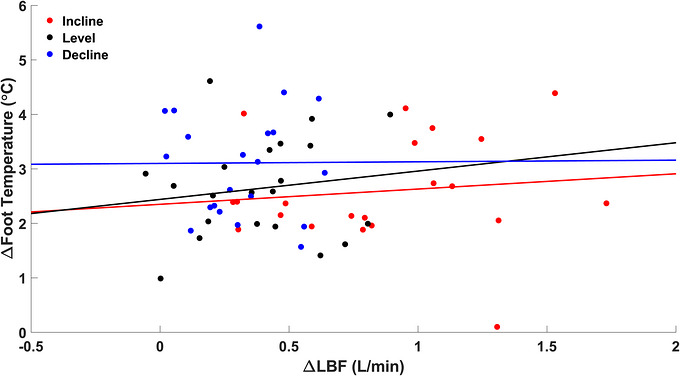
The association between the change in leg blood flow (LBF; *x*‐axis) and the change in foot temperature (*y*‐axis) in all walking conditions. There was no significant relationship between changes in LBF and foot temperature during walking (*P* = 0.543).

The second LMM showed that changes in LBF and baseline foot temperature did not contribute significantly to changes in foot temperature (Table 2; Figure 4). These findings suggest that LBF and pre‐walking foot temperature do not influence the increase in foot temperature during walking.

**TABLE 2:** Estimates of the influence of changes in leg blood flow and baseline foot temperature on changes in foot temperature.

Predictor	Regression coefficient	*P*‐value	95% Confidence interval
Intercept	4.624	0.200	−2.574 to 11.822
ΔLeg blood flow	−0.193	0.543	−0.825 to 0.439
Baseline foot temperature	0.072	0.624	−0.369 to 0.225

Dependent variable: Δfoot temperature.

**FIGURE 5:**
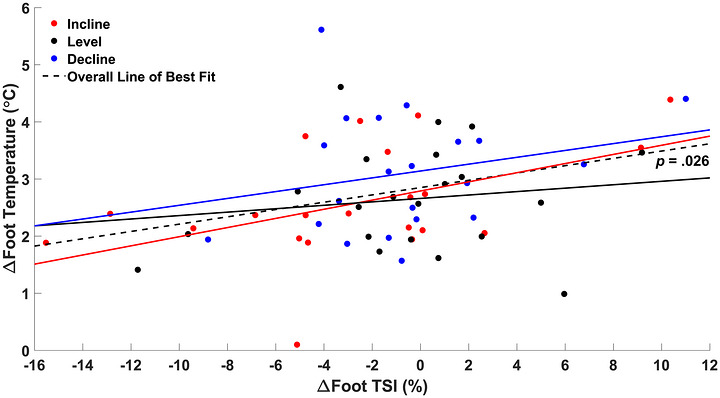
The association between the change in dorsal foot tissue saturation index (TSI; *x*‐axis) and the change in foot temperature (*y*‐axis) in all walking conditions. The dotted black line is the regression line formulated from linear mixed‐model results in Table 3 (*P* = 0.026).

The third LMM showed that changes in leg muscle TSI and baseline foot temperature did not contribute significantly to changes in foot temperature (Table 3; Figure 5). However, changes in DF TSI were associated with changes in foot temperature (*P* = 0.026), suggesting that changes in DF TSI might influence changes in foot temperature.

**TABLE 3:** Estimates of influence of changes in leg muscle tissue saturation index and baseline foot temperature on changes in foot temperature.

Predictor	Regression coefficient	*P*‐value	95% Confidence interval
Intercept	1.292	1.00	−5424.636 to 5427.220
ΔVL TSI	0.037	0.519	−0.077 to 0.150
ΔMG TSI	0.028	0.192	−0.014 to 0.070
ΔDF TSI	0.064	0.026	0.008 to 0.120
Baseline foot temperature	0.065	0.650	−0.225 to 0.356

Dependent variable: Δfoot temperature.

Abbreviations: DF, dorsal foot; MG, medial gastrocnemius; TSI, tissue saturation index; VL, vastus lateralis.

## 4 DISCUSSION

This study was a non‐invasive investigation of the effects of macro‐ and microcirculatory factors on the change in foot temperature during walking on different slopes. Our first hypothesis was supported, in part, because uphill walking caused the greatest increase in LBF and oxygen consumption (i.e., reduction in TSI) in the VL and MG muscles compared with level walking, but oxygen consumption did not change in the foot dorsum across all conditions. Our second hypothesis was not supported, because changes in LBF and leg muscle TSI were not predictive of the change in foot temperature. In other words, we observed a concurrent increase in LBF and reduction in VL and MG TSI, whereas there was no association between LBF and foot temperature change as a function of walking. These results suggest that the macrocirculatory response (increase in LBF) is not related to changes in foot skin temperature but instead is likely to be related to the oxygen demands of the leg skeletal muscles during walking.

We speculate that the null relationship between macrocirulatory changes and foot temperature (Figure 4) could be attributable to a small percentage of total LBF that reaches the foot muscles during walking. In guinea fowl, for example, the largest knee extensor (iliotibialis lateralis pars postacetabularis) and ankle plantarflexor (gastrocnemius medialis and lateralis) experience a greater increase in blood flow compared with the largest foot muscles (deep digital flexors) during running (Rubenson et al., 2006). If similar redistribution of blood flow exists in humans, it is possible that the blood reaching the foot might not be enough to cause a significant change in foot temperature. The redistribution of blood could be driven by differences in the oxygen demand of the individual leg muscles during walking. We found that walking on a 5° incline causes greater oxygen consumption in the VL and MG muscles but not in the foot dorsum (Figure 3). Previous research has also found that mechanical work done by the knee and ankle joints increases significantly as walking slope increases, whereas work done by the foot does not (Papachatzis & Takahashi, 2023). Likewise, muscle activity and force produced by the knee extensors and ankle plantarflexors also increase significantly when walking on an incline compared with a level surface (Alexander & Schwameder, 2016; Franz & Kram, 2013). Altogether, these findings suggest that the workload of the human knee extensor and ankle plantarflexor increases when walking uphill, leading to increased muscle oxygen consumption and a greater increase in LBF compared with level walking. A lower rate of oxygen consumption at the foot probably caused a small increase in blood flow to the foot in comparison to the VL and MG muscles. This small increase in foot blood flow alone might not be sufficient to increase foot temperature significantly during walking. Instead, changes in the downstream microcirculation within the tissues of the foot could explain changes in foot temperature during walking.

Changes in microcirculation at the VL and MG muscles were not associated with foot skin temperature change, in contrast to changes in the microcirculation at the dorsal foot (Table 3). Null relationships between thigh and calf muscle oxygen consumption and foot temperature change could be attributed to the distance between the muscles and the foot. It is unlikely that any heat generated by the thigh and calf muscles through oxygen uptake and metabolism would propagate distally and increase foot temperature. Metabolism at the foot dorsum is more likely to influence foot temperature during walking owing to its proximity to the foot plantar surface (Figure 5).

We observed a positive association between the change in dorsal foot TSI and the change in foot temperature (*P* = 0.026); however, the direction of correlation was contrary to our expectations. We expected a negative correlation between the change in dorsal foot TSI and foot temperature change, because a decrease in TSI could indicate increased oxygen consumption, thereby generating heat and increasing foot temperature. Although this finding was contrary to our expectations, we speculate that it might hint at the role of microcirculatory blood flow in plantar temperature change during walking in several ways. First, as blood flow is redistributed to the foot during walking via microvascular dilatation (M. L. Armstrong et al., 2007; Bagher & Segal, 2011; Casey & Joyner, 2012; Casey et al., 2013; Clifford & Hellsten, 2004; Crecelius et al., 2013; Credeur et al., 2015; Dinenno, 2016; Hong & Kim, 2017; Naik et al., 1999; Stamler & Meissner, 2001), venous return from the legs increases via the skeletal muscle pump mechanism (Kügler et al., 2001; Tauraginskii et al., 2023, 2024), possibly resulting in less deoxygenated blood in the dorsal foot tissue. Simultaneously, the presumably small amount of oxygenated blood that is redistributed to the dorsal foot might not be completely used by muscles and soft tissues and could result in greater oxygen saturation at the dorsal foot. If the same process happens in the muscle and soft tissue on the bottom of the foot, the increased venous return and arterial blood flow, which increase dorsal foot oxygen saturation, could simultaneously increase temperature despite the oxygen supply is not being fully utilized. Such a finding would support the idea that foot temperature change could be attributed not only to the mechanical forces exerted on the plantar surface during walking, but also to physiological processes that occur simultaneously (Burnett et al., 2025). Future studies could measure how arterial and venous blood flow change with walking at the foot, in order to elucidate the mechanisms underpinning this finding. Second, we speculate that any increase in skin temperature caused by increased oxygen consumption at the foot dorsum (i.e., reduction in TSI) might be masked by physiological cooling mechanisms. Increased skin temperature at the foot could proportionally increase cutaneous vasodilatation (Bornmyr et al., 1997; Del Pozzi & Hodges, 2015; Del Pozzi et al., 2013, 2016; Wu et al., 2017) and sweating (Cramer & Jay, 2015; Gagnon et al., 2013; Ravanelli et al., 2020) to attenuate temperature accumulation during walking, resulting in a smaller increase in temperature as oxygen consumption increases. Alternatively, there might be other mechanisms external to the body that could be influencing the change in foot temperature in our experiment.

We speculate that there are several mechanisms that influence the change in foot temperature during walking that were not considered in the present study. For instance, previous research has found significant positive correlations between foot temperature change and shear forces (i.e. frictional forces) exerted on the foot by the ground during walking (Gonzalez et al., 2021; Yavuz et al., 2014, 2015). These frictional forces vary on different walking slopes, with sloped walking eliciting greater mechanical strain (Crossland et al., 2023) and anteroposterior ground reaction forces (Gottschall & Kram, 2005; Lay et al., 2006; McIntosh et al., 2006) in comparison to level walking. Thus, it could be hypothesised that walking on a slope would increase foot temperature the most. Yet, our results show that foot temperature increased to a similar extent during each walking condition (Figure 1). Therefore, there must be other factors that regulated foot temperature during walking in our experiment that we did not measure, such as heat convection, sweating and skin microvascular dilatation. Participants wore shoes that exposed the entire dorsal surface of the foot, thereby facilitating heat transfer to the air and aiding foot skin temperature regulation during walking. Participants’ feet might also have begun to sweat during each walking condition, allowing evaporative heat loss to occur. Microvascular dilatation in the skin could also increase heat dissipation from the skin to the air (Rowell, 1974, 1977). As blood vessels dilate, their surface area will increase exponentially, thus improving heat dissipation from the skin to the air by increasing the heat transfer coefficient of the skin (Liang et al., 1991). The abovementioned factors might act simultaneously to change skin temperature during walking, resulting in similar changes in plantar temperature during various walking intensities. However, in this study we did not quantify the contributions of these other mechanics of foot temperature change. Future research should measure these other factors simultaneously to create a more coherent picture of foot temperature change.

Limitations of this study include the absence of additional measurements, including indirect calorimetry, body composition, LBF measures during walking, TSI on a plantar surface and measures of sweating, in addition to short walking bouts. The aerobic capacity and lean muscle mass of participants are factors that can influence vascular function and leg muscle oxygen consumption (Rådegran & Saltin, 1998, 2000), although we would expect that these limitations would not affect the outcomes of our study owing to our repeated‐measures design. The deformation of the common femoral artery during walking made Doppler ultrasound measurements difficult to obtain. To circumvent this limitation, we measured LBF during quiet standing immediately before and after each walking condition using a 3D printed bracket that fastened over the participant's common femoral artery while they walked. Fastening the bracket to the participant allowed us to record ultrasound measurements within seconds after the end of walking bouts to capture the most accurate hyperaemic responses to walking possible. We also could not measure plantar foot TSI during walking because of concerns about participant comfort and the risk of damage to equipment. Instead, we recorded TSI at the dorsal surface of the foot, because it was the most feasible location on the foot to place the NIRS probe. However, dorsal foot TSI might not accurately reflect plantar foot TSI, which might be better associated with plantar foot temperature change during walking. Future research could standardize NIRS probe placement on the plantar surface of the foot to record pre‐ and post‐walking plantar foot TSI. Finally, studies have shown that the rate of increase of foot temperature depends on the duration of walking bouts, with foot temperature increasing linearly at the onset of walking, then plateauing at ∼33°C after ∼20 min of walking (Burnett et al., 2025; Reddy et al., 2017). Our walking bouts were only 10 min long, which would not allow us to determine whether the measurements made in the present study are associated with the plateau timing and temperature.

## 5 CONCLUSION

In conclusion, we showed that walking on a slope influences changes in leg macrocirculation and microcirculation in regions proximal to the foot. However, vascular‐related changes proximal to the foot were not predictive of a change in foot temperature after 10 min walking bouts on various slopes. Instead, the microcirculatory changes occurring at the foot might contribute to the change in foot temperature brought about by walking. These findings suggest that LBF does not affect foot skin temperature change. Instead, LBF most probably increases to meet the metabolic demand of the leg muscles that enable walking. Those with cardiovascular diseases might not have the capacity to meet the metabolic or vascular demands of walking, hence the findings of this study cannot be generalized to clinical populations. Future investigations could use our framework to investigate the contribution of vascular dysfunction to skin temperature change in those with cardiovascular disease.

## AUTHOR CONTRIBUTIONS

All authors conceived and designed research; Jose G. Anguiano‐Hernandez and Jenna K. Burnett performed experiments. Jose G. Anguiano‐Hernandez analysed data. All authors interpreted results of experiments. Jose G. Anguiano‐Hernandez prepared figures and drafted the manuscript. All authors edited and revised the manuscript. All authors approved the final version of manuscript and agree to be accountable for all aspects of the work in ensuring that questions related to the accuracy or integrity of any part of the work are appropriately investigated and resolved. All persons designated as authors qualify for authorship, and all those who qualify for authorship are listed.

## CONFLICT OF INTEREST

None declared.

## GENERATIVE AI STATEMENT

No generative AI tools were used in the preparation of this manuscript.

## Data Availability

The datasets used and/or analysed during the present study are available from the corresponding author on reasonable request.
